# Prion disease modelled in *Drosophila*

**DOI:** 10.1007/s00441-022-03586-0

**Published:** 2022-01-29

**Authors:** Raymond Bujdoso, Andrew Smith, Oliver Fleck, John Spiropoulos, Olivier Andréoletti, Alana M. Thackray

**Affiliations:** 1 Department of Veterinary Medicine, University of Cambridge, Madingley Road, Cambridge, CB3 OES UK; 2 Pathology Department, Animal and Plant Health Agency (APHA), Weybridge, Woodham Lane, New Haw, Surrey KT15 3NB Addlestone, UK; 3 UMR INRA ENVT 1225-Hôtes-Agents Pathogènes, Ecole Nationale Vétérinaire de Toulouse, 23 Chemin des Capelles, 31076 Toulouse, France

**Keywords:** *Drosophila*, Infectivity, Neurodegeneration, Prion, Transmissible

## Abstract

Prion diseases are fatal neurodegenerative conditions of humans and various vertebrate species that are transmissible between individuals of the same or different species. A novel infectious moiety referred to as a prion is considered responsible for transmission of these conditions. Prion replication is believed to be the cause of the neurotoxicity that arises during prion disease pathogenesis. The prion hypothesis predicts that the transmissible prion agent consists of PrP^**Sc**^, which is comprised of aggregated misfolded conformers of the normal host protein PrP^**C**^. It is important to understand the biology of transmissible prions and to identify genetic modifiers of prion-induced neurotoxicity. This information will underpin the development of therapeutic and control strategies for human and animal prion diseases. The most reliable method to detect prion infectivity is by in vivo transmission in a suitable experimental host, which to date have been mammalian species. Current prion bioassays are slow, cumbersome and relatively insensitive to low titres of prion infectivity, and do not lend themselves to rapid genetic analysis of prion disease. Here, we provide an overview of our novel studies that have led to the establishment of *Drosophila melanogaster*, a genetically well-defined invertebrate host, as a sensitive, versatile and economically viable animal model for the detection of mammalian prion infectivity and genetic modifiers of prion-induced toxicity.

## Introduction

Prion diseases are fatal and transmissible neurodegenerative diseases of the CNS, which include Creutzfeldt-Jakob disease (CJD) of humans, bovine spongiform encephalopathy (BSE) in cattle, chronic wasting disease (CWD) of cervids and scrapie in sheep (Prusiner 2004). A central feature of prion disease pathogenesis is the accumulation of PrP^**Sc**^, an abnormal conformer of the host protein PrP^**C**^, in the brains of affected individuals (Bolton et al. 1982). The prion hypothesis proposes that a novel proteinaceous moiety termed a prion is responsible for transmission of these diseases (Prusiner 1982). Significant evidence suggests that prions comprise principally, if not solely, of PrP^**Sc**^ since prions that are transmissible in vivo can be generated from recombinant PrP in vitro (Legname et al. 2004; Wang et al. 2010).

Prions lack a nucleic acid-based genome and do not stimulate a typical immune response in those hosts infected with these novel transmissible agents (Prusiner 2004). Consequently, prions are not detected by commonly used techniques that identify conventional pathogens, such as viruses and bacteria. Instead, in vitro techniques can be used to identify the surrogate marker of transmissible prions, namely PrP^**Sc**^. Western blot can be used to detect proteinase K (PK)-resistant PrP^**Sc**^ (Schaller et al. 1999) while immunoassays can be used to detect both PK-resistant and PK-sensitive PrP^**Sc**^ (Safar et al. 1998; Thackray et al. 2007). In addition, in vitro amplification of PrP^**Sc**^, mediated by prion-seeding activity, can be achieved by protein misfolding cyclic amplification (PMCA) and RT-QuIC (Saborio et al. 2001; Wilham et al. 2010). These biochemical-based procedures are used on the premise that the amount of PrP^**Sc**^ correlates with the degree of prion infectivity (Beekes et al. 1996; Bolton et al. 1991; Jendroska et al. 1991). However, the level of PrP^**Sc**^ and prion infectivity are not always congruent (Lasmezas et al. 1997; Miyazawa et al. 2011), and as a consequence, in vivo bioassay in a suitable experimental host remains the most reliable method for the detection of bona fide prion infectivity.

Prion bioassays have been used to measure the amount of prion infectivity in different tissues and fluids derived from prion-infected individuals. The goal of these studies is to understand the biology of infectious prions and to ensure the safety of animal products destined for human consumption, since animal prion diseases are a significant public health risk through their potential for zoonotic transmission. This risk has been realised with the identification of variant CJD (vCJD) in humans as a consequence of the consumption of prion-contaminated bovine products. The discovery of new prion diseases, such as atypical scrapie in sheep (Benestad et al. 2003), atypical BSE in cattle (Beringue et al. 2006; Biacabe et al. 2004) and camelid prion disease (Babelhadj et al. 2018), and the emergence of new reservoirs of CWD in Europe (Benestad et al. 2016) present unidentified zoonotic risks to human health. In addition to their role in public health, prion transmission studies are used to probe the molecular basis of prion propagation and the transmission barrier that controls their passage between species (Prusiner 2004), and to help provide an understanding of the cellular and molecular mechanisms of prion-induced neurotoxicity.

Prion diseases are difficult to study under natural conditions as the exact time of prion exposure cannot be determined and the full course of the disease cannot be analysed. As a consequence, prion diseases are modelled in experimental animal hosts. To date, a variety of different mammalian species, ranging from large experimental animals, including non-human primates, to small rodents, have been used as hosts in prion transmission studies (Brandner and Jaunmuktane 2017). However, prion studies that rely on mammalian species as the experimental host have practical disadvantages since they take a considerably long time, are expensive, do not readily detect low levels of prion infectivity and are subject to increasing ethical debate. Here, we describe our studies that have established *Drosophila melanogaster* as a more sensitive, economically viable host for the relatively rapid detection of mammalian prions and one that lends itself to the search for genetic modifiers of prion-induced neurotoxicity.

### Mammalian species as hosts in prion transmission studies

Developments in prion biology have been inextricably linked to involvement of a wide range of animal species used to detect prion infectivity, including large mammals (Brandner and Jaunmuktane 2017). The first reported transmission of a prion disease was by Cullié and Chelle in 1936 who inoculated brain and spinal cord from a scrapie-affected sheep into healthy same-species recipients by various routes including intraocular administration (Cullié and Chelle 1936). Early attempts to identify the molecular nature of the transmissible agent were performed using fractionated scrapie-infected ovine brains and bioassays in sheep (Gordon 1946; Pattison and Millson 1960; Stamp et al. 1959). BSE-infected blood transfusion experiments have been performed in sheep in order to model blood-borne vCJD transmission in humans (Hunter 2003; Salamat et al. 2021). A variety of non-human primates, including chimpanzees, have been inoculated with brain material from human prion disease in order to establish these conditions were transmissible (Gajdusek et al. 1966). Cynomolgus macaques inoculated with brain extracts from classical BSE-infected cattle produced a pathological phenotype that was almost identical to vCJD, which provided evidence to establish this bovine prion disease as a zoonotic (Lasmezas et al. 1996). Old World and New World monkeys have collectively been used to investigate the zoonotic potential of other animal prion diseases including scrapie (Comoy et al. 2015; Gibbs et al. 1980; Gibbs and Gajdusek 1972), atypical BSE (Comoy et al. 2008) and CWD (Marsh et al. 2005; Race et al. 2018). In addition to these prion transmission studies, experimental pathogenesis studies of scrapie, BSE and CWD have been performed in sheep, cattle and cervids, respectively (Orge et al. 2021).

Small rodents including mice, hamsters and bank voles have been used extensively as experimental models of prion disease (Groschup and Buschmann 2008). Chandler was the first to transmit mammalian prions to rodents through the successful inoculation of scrapie-infected goat brain material into wild-type mice (Chandler 1961). Serial transmission of sheep scrapie through inbred mouse lines allowed adaptation and characterisation of different prion strains (Chandler 1961; Fraser and Dickinson 1968). Hamsters were used as the experimental bioassay host in landmark experiments that identified PrP^**Sc**^ as the biochemical component that co-purified with prion infectivity following fractionation of scrapie-infected hamster brains (McKinley et al. 1983). Bank voles have been identified as a host permissive for facilitated transmission of prions from multiple different species (Cartoni et al. 2005; Nonno et al. 2006).

Inoculation of wild-type mice and hamsters with different species’ forms of prions helped establish the phenomenon of the transmission barrier whereby successful passage between species is generally less efficient compared to propagation within their natural hosts (Pattison 1965). Inefficient attack rates and prolonged incubation times at primary passage are indicative of a prion transmission barrier, whereas enhanced attack rates and shortened incubation times are observed following serial passage in hosts that express the same PrP^**C**^ amino acid sequence (Hill et al. 2000). Abrogation of the species barrier has been achieved in mice with the endogenous murine PrP gene inactivated and that overexpress a PrP transgene homologous to the host from which the prion isolates were derived (Brandner and Jaunmuktane 2017; Moreno and Telling 2017). This has led to a plethora of different mouse models that allow facilitated transmission of prions, including those of sheep, bovine, human and cervid origin, in mice transgenic for PrP genes from these different species.

Despite their considerable importance, prion transmission studies in mammalian species are problematic. Those using large experimental animals are hampered by long incubation times prior to the onset of clinical prion disease and significant practical difficulties and large financial costs associated with housing these species. Collectively, these issues result in constraints on the number of large animals used in each experiment with subsequent loss of statistical power. While small rodents are less cumbersome to maintain and generally show shorter incubation times than large experimental animals, prion transmissions in PrP transgenic mice may take many months or even years to complete and show only a 1–2 log LD_**50**_ higher infectivity titre than the respective homologous species (Groschup and Buschmann 2008). In addition, there is increasing ethical debate about the use of mammalian hosts to bioassay prions, in particular non-human primates. For these reasons, it is important to develop more efficient bioassays to assess mammalian prion infectivity, preferably using a less sentient host.

### *Drosophila melanogaster* as a versatile experimental animal system

New animal models of mammalian prion diseases should allow sensitive and relatively rapid detection of prion infectivity, ideally in a genetically well-defined animal system that enables analysis of genes and metabolic pathways involved in prion neurotoxicity. The successful application of PrP transgenesis in rendering xenogeneic hosts susceptible to different species forms of prions (Crozet et al. 2001; Vilotte et al. 2001) provides an opportunity to explore the development of non-mammalian animal models of prion disease in pursuit of these goals.

*Drosophila melanogaster* has emerged as a model system for the study of complex biological processes, such as mammalian neurodegenerative disease (Lu and Vogel 2009). This arises for several reasons: (i) the brains of *Drosophila* and mammalian species are composed of similar components (i.e. neurons and neuronal circuitry), and the nature of ion channels, neurotransmitters and synaptic proteins are highly conserved between mammals and the fly (Lessing and Bonini 2009); (ii) *Drosophila* is a genetically well-defined organism that lends itself to the production of transgenic flies that express proteins in a tissue-specific distribution; (iii) the normal physiology and development of *Drosophila* is well characterised so that phenotypic assays can readily detect neurotoxicity in the live organism (Marsh and Thompson 2006); and (iv) large numbers of flies can be produced in a short time, which correlated with their relatively short life span (Piper et al. 2005), allows rapid, statistically robust data collection.

**Fig. 1:**
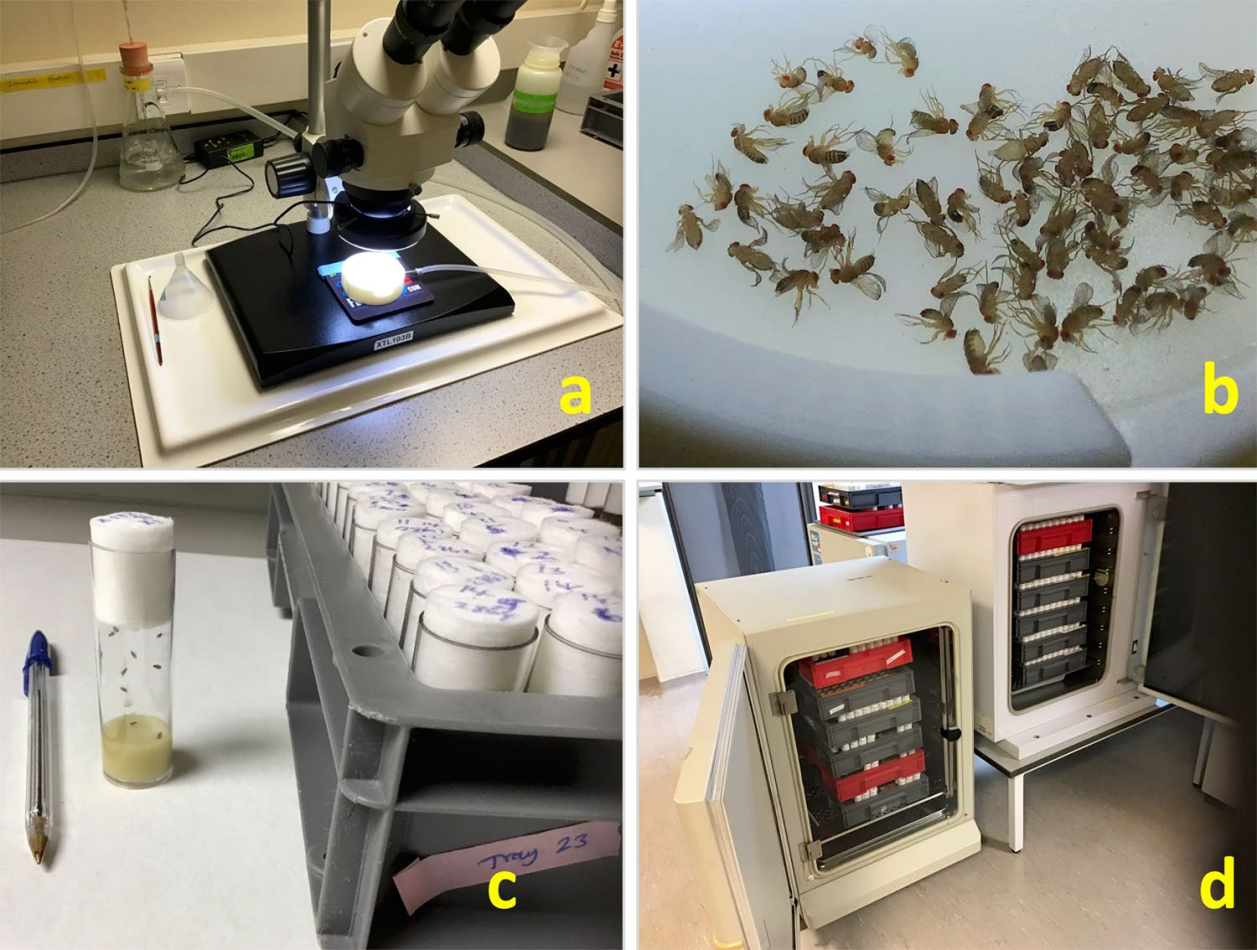
Some general aspects of *Drosophila* husbandry. (**a**) Phenotypic sorting of *Drosophila*, or ‘fly pushing’, requires the use of a stereomicroscope; (**b**) *Drosophila* are anesthetised using CO_2_ prior to sorting; (**c**) *Drosophila* are housed in vials and raised on cornmeal media; and (**d**) prion-inoculated *Drosophila* are maintained at 25 °C in incubators

*Drosophila* have already proved to be an important animal model to analyse the genetics of mammalian neurodegeneration. For example, the *Drosophila* gene *amyloid precursor protein-like (Appl)* gene encodes a protein product (APPL) (Rosen et al. 1989) that is orthologous to the human amyloid beta precursor protein (APP) associated with Alzheimer’s disease (Kang et al. 1987). Deletion of *Appl* in *Drosophila* causes behavioural defects that can be compensated for by transgenic expression of the human gene APP (Luo et al. 1992). In addition, mutagenesis screens in *Drosophila* have led to the isolation of flies that show late-onset progressive degeneration of the adult nervous system that resembles human diseases (Min and Benzer 1999). Significantly, *Drosophila* have been used to model a number of human neurodegenerative diseases including Parkinson’s disease (Auluck et al. 2002; Feany and Bender 2000) and tauopathies (Fulga et al. 2007; Wittmann et al. 2001) such as Alzheimer’s disease (Greeve et al. 2004; Iijima et al. 2004). For these reasons, we considered that *Drosophila melanogaster* could be an ideal animal system for development as an invertebrate model of mammalian prion disease. Some general aspects of *Drosophila* husbandry are highlighted in Fig. 1.

### *Drosophila* efficiently express mammalian PrP

*Drosophila melanogaster* and other members of the arthropod phylum do not show the presence of an orthologous PrP gene in their genome and therefore do not normally express PrP^**C**^ (Rivera-Milla et al. 2006). Only hosts that express PrP^**C**^ are susceptible to prion-induced toxicity, and formation of the toxic moiety occurs concomitantly with prion replication (Bueler et al. 1993; Mallucci et al. 2003). Consequently, it is necessary for *Drosophila* to be rendered transgenic for PrP expression in order to be permissive for mammalian prion propagation.

Several groups have shown the expression of mammalian PrP in *Drosophila.* The first report of PrP transgenic *Drosophila* was by Raeber et al. who expressed wild-type hamster prion protein under the control of the fly hsp70 gene promoter (Raeber et al. 1995). Hamster PrP expressed in *Drosophila* exhibited variable glycosylation and the presence of a glycosylphosphatidyl inositol (GPI) anchor, features reminiscent of those seen in the native host. PrP expressed in *Drosophila* showed a lower molecular mass compared to expression in mammalian species. This arises because in *Drosophila*, post synthetic trimming reactions of *N*-linked glycosylation moieties result in a lack of sialylation in contrast to the complex sugar groups that are added to proteins in mammalian systems (Marchal et al. 2001; März et al. 1995; Repnikova et al. 2010). Subsequent studies by others showed that *Drosophila* transgenic for wild-type hamster or mouse PrP was characterised by locomotor dysfunction, as well as degeneration and vacuolation in the fly brain (Fernandez-Funez et al. 2009, 2010). Locomotor dysfunction in murine PrP transgenic *Drosophila* was mitigated by mutations in the α-helix 1-β-strand 2 loop (Sanchez-Garcia et al. 2016) and in α-helix 3 (Sanchez-Garcia et al. 2013). Expression of Val129 human PrP in *Drosophila* was reported to induce a toxic eye phenotype that was exacerbated when PERK, a component of the unfolded protein response, was overexpressed at the same time (Fernandez-Funez et al. 2017). However, Park et al. determined that the unfolded protein response did not appear to be activated in *Drosophila* transgenic for murine PrP (Park et al. 2011).

Several groups have reported the generation of *Drosophila* transgenic for expression of mammalian PrP harbouring mutations associated with genetic prion disease. Deleault et al. expressed mouse PrP in the fly that contained additional *N*-terminal octapeptide repeat inserts, a mutation associated with familial CJD, which did not induce a toxic fly phenotype (Deleault et al. 2003). *Drosophila* transgenic for mouse PrP containing the P101L mutation, which is associated with GSS in humans, developed a toxic phenotype, that included larval lethality and locomotor dysfunction and reduced survival in adult flies, that was not seen in *Drosophila* that expressed wild-type mouse PrP (Gavin et al. 2006). However, expression of the P101L PrP in *Drosophila* neurons was subsequently determined to be more than 20 times greater than that of wild-type PrP (Gavin et al. 2008). Analysis of further *Drosophila* fly lines with expression of P101L mouse PrP showed that the action potential frequency of neurons, as well as fly locomotor ability and survival, was reduced in mutant mouse PrP transgenic *Drosophila* relative to flies that expressed wild-type mouse PrP (Choi et al. 2010; Gavin et al. 2006; Murali et al. 2014). Meanwhile, Robinson et al*.* showed that flies which expressed 3F4 epitope wild-type mouse PrP had synaptic changes, including increases of miniature excitatory junctional currents, synaptic vesicle sizes and vesicle release probability (Robinson et al. 2014). However, these changes were less pronounced in flies that expressed P101L mouse PrP, which suggested that the mutated PrP was less functional at synapses compared to wild-type PrP.

While the aforementioned studies of PrP transgenic *Drosophila* have been informative, they also have their shortcomings. In the majority of cases, the PrP transgenic *Drosophila* described above were generated by random incorporation of the transgene into the fly genome. This has the potential for mutational positional effects on either PrP expression or endogenous gene expression, or multiple transgene copy number, thereby complicating comparisons between different fly lines. Some of the studies reported spontaneous toxic phenotypes in *Drosophila* that expressed either wild-type or mutated forms of PrP. However, none of these studies examined if this was a transmissible toxicity, either by fly-to-fly or fly-to-mammalian host transmission, in order to demonstrate that the effect was prion-mediated. In addition, some of the studies, including those that investigated wild-type PrP expression in *Drosophila*, reported reactivity of transgenic fly material with monoclonal antibody 15B3, a putative disease-associated PrP-specific reagent. However, these 15B3 observations were not supported with either the biochemical detection of proteinase K-resistant PrP by SDS PAGE/western blot or detection of PMCA prion-seeding activity, which are both well-established methods for the detection of PrP^**Sc**^, the surrogate marker of transmissible prions. Therefore, it is reasonable to conclude that these early studies clearly showed that mammalian PrP could be expressed in *Drosophila*, none of the studies addressed whether any of the observed phenotypic or biochemical responses were due to effects of *bona fide* transmissible prions.

### Prion disease modelled in *Drosophila*

We modelled classical scrapie of sheep in the fly in order to test the hypothesis that PrP transgenic *Drosophila* are susceptible to prion-induced toxicity and permissive for mammalian prion propagation. Classical scrapie, the prototypic prion disease, is a naturally occurring acquired condition of sheep and other small ruminants that exhibits strain diversity (Hunter 1997). Sheep that express the ovine PrP variant V^136^R^154^Q^171^ (termed VRQ, where V, R and Q stand for valine, arginine and glutamine, respectively) or A^136^R^154^Q^171^ (termed ARQ, where A stands for alanine) are susceptible to classical scrapie (Clouscard et al. 1995; Goldmann et al. 1994). Furthermore, facilitated transmission of classical scrapie occurs in hosts rendered transgenic for these ovine PrP variants (Crozet et al. 2001; Thackray et al. 2008; Vilotte et al. 2001). In this context, we tested the hypothesis that *Drosophila* transgenic for VRQ or ARQ ovine PrP would be susceptible to classical scrapie infection.

**Fig. 2:**
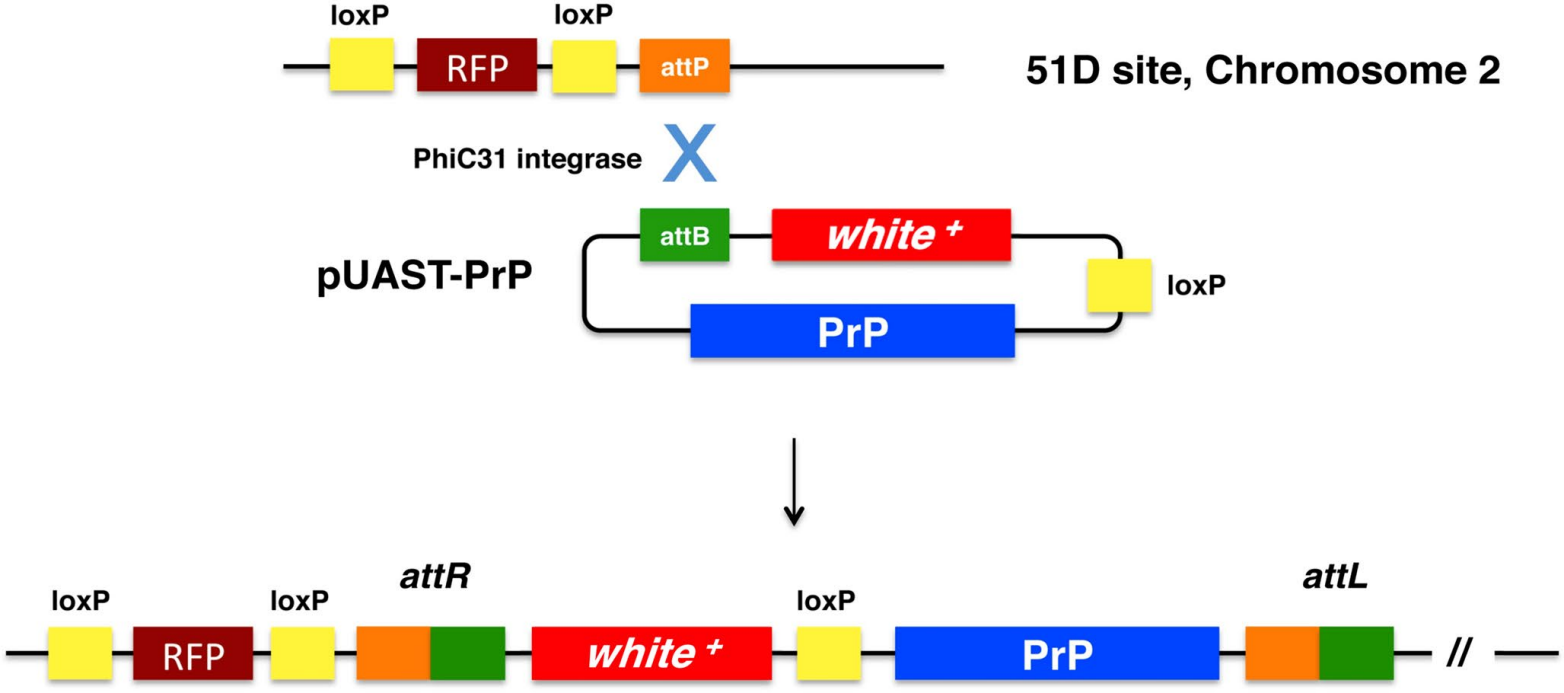
PhiC31/pUAST-mediated site-specific transgenesis in *Drosophila*. The pUASTattB-PrP plasmid contains an *attB* fragment, the *white*^+^ marker gene and the PrP transgene. During pUAST/PhiC31 integrase-mediated integration, the unique *attP* site in the *ZH-attP-51D* fly line genome recombines with the pUAST *attB* site in the pUAST-PrP vector forming *attR* and *attL* sites in the generated transgenic flies (Bischof et al. 2007). This results in the integration of pUASTattB-PrP DNA into the fly genome, thereby creating *attR* and *attL* hybrid sites. The *loxP* sites allow the elimination of the red fluorescence protein (RFP) and *white*^+^ marker genes after PhiC31-mediated transgenesis. (Taken from Thackray et al., *Biochem. J*., 2014a, 463: pp31-40, supplementary data)

We generated *Drosophila* transgenic for VRQ and ARQ ovine PrP through the site-specific pUASTattB/PhiC31 integrase system, which delivers a single copy of the transgene of interest into a defined landing pad in the fly genome (Bischof et al. 2007) as outlined in Fig. 2. The PrP transgenes comprised DNA encoding mature length ovine PrP (amino acid residues 25–232) flanked by the coding sequences for an insect leader sequence peptide (Green et al. 2000) at the 5′ end and the ovine GPI anchor sequence at the 3′ end. The PrP constructs were ligated into the *Drosophila* integration vector pUASTattB for injection into embryos of the 51D fly line. Stable balanced *UAS-*ovine PrP transgenic *Drosophila* were generated by conventional fly crossing and crossed with the *elav*-GAL4 driver fly line to stimulate pan neuronal PrP expression.

**Fig. 3:**
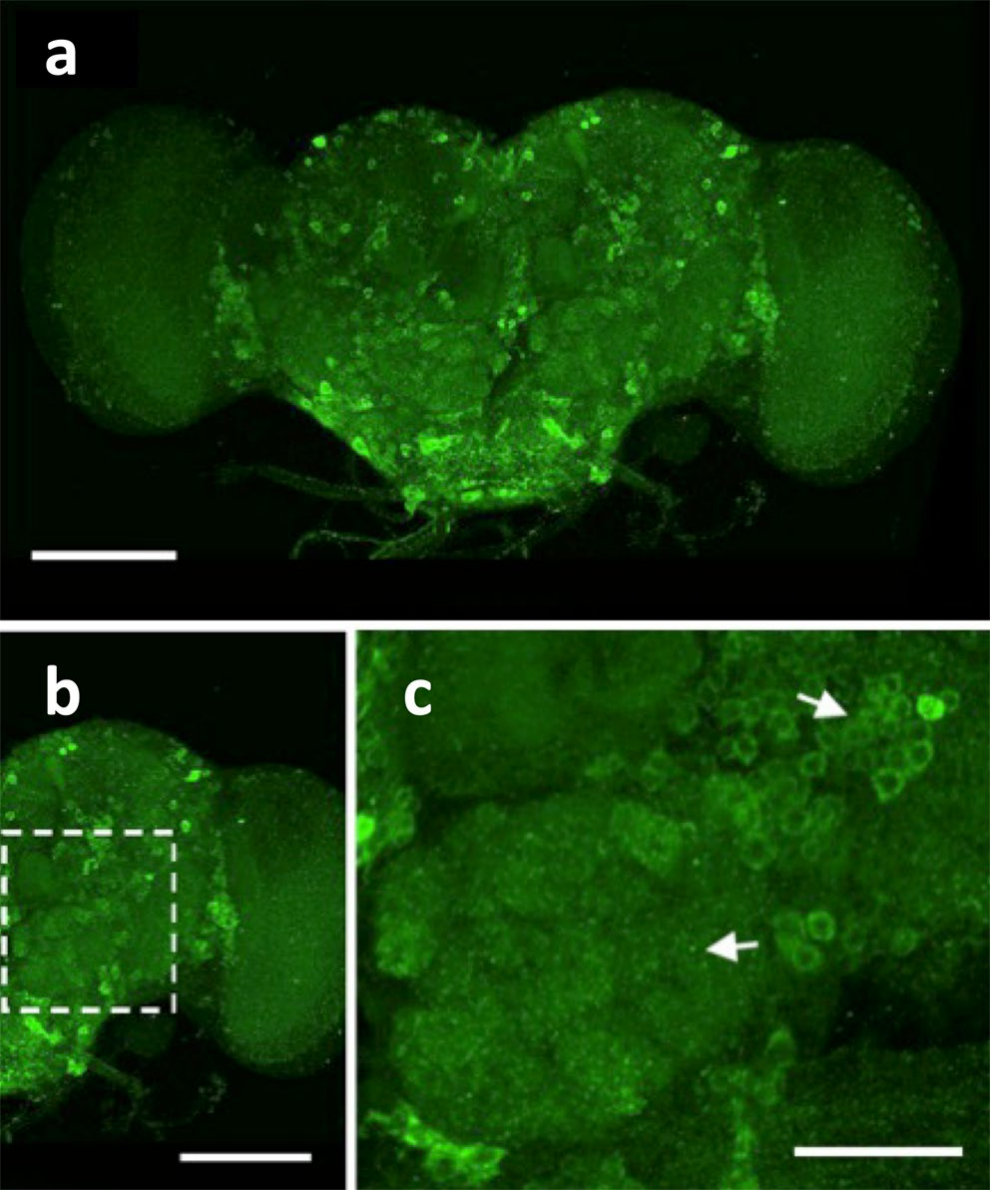
Distribution of prion protein in the brain of ovine PrP transgenic *Drosophila*. Adult (5-day-old) *Elav* > ARQ ovine PrP *Drosophila* fly brains were isolated and stained with anti-PrP monoclonal antibody 6H4 detected by Alexa Fluor 488-labelled goat anti-mouse IgG. (**a**) Whole brain, (**b**) section of whole brain with an enlargement of the dashed area in (**c**) that shows PrP expression associated with neuronal cell bodies (upper arrow) and antennal lobe glomeruli (lower arrow). Scale bars 100 nm in (**a**) and (**b**) and 40 nm in (**c**). (Modified from Thackray et al., *Biochem. J*., 2012b, 444: pp487-495)

Western blot analysis showed that ovine PrP expressed in *Drosophila* had a molecular mass of 29–31 kDa and comprised at least two major protein bands, and a third less intense band, similar to that of other species forms of PrP expressed in *Drosophila* (Thackray et al. 2012b). Prion protein expression was also evident associated with neuronal cell bodies of the ovine PrP fly lines as shown in Fig. 3. Adult fly brains exhibited occasional vacuolar lesions of variable size that were distributed randomly throughout ovine PrP *Drosophila* and control 51D flies that lack PrP expression. This indicated that the detected vacuoles were artefactual and that overexpression of mammalian PrP in *Drosophila* alone does not adversely affect neuronal integrity or mediate neurodegeneration.

We next exposed *Drosophila*, with pan neuronal ovine PrP expression, to exogenous prions in order to determine whether these flies were susceptible to prion-induced neurotoxicity. To do so, fly food was supplemented with scrapie-infected sheep brain homogenate to allow oral exposure of VRQ ovine PrP *Drosophila* larvae to ovine prions (Thackray et al. 2012a), which mimicked what is considered to be the natural route of prion infection in most cases of transmissible mammalian prion disease (Glatzel and Aguzzi 2000; Mabbott and MacPherson 2006), including classical scrapie of sheep (van Keulen et al. 2008). Remarkably, after hatching, prion-exposed adult PrP *Drosophila* showed an accelerated decline in locomotor ability that became progressively more severe with age and displayed a shortened survival time compared to similar flies fed control prion-free sheep brain material (Thackray et al. 2014a, b, 2012a). These neurotoxic phenotypes were PrP-dependent and prion-specific since they were only induced in PrP transgenic *Drosophila* and only after exposure to scrapie inoculum.

### Mammalian prions replicate in *Drosophila*

In mammalian species, clinical signs in prion affected individuals arise through neurodegenerative processes initiated by the conversion of PrP^**C**^ to PrP^**Sc**^. Accordingly, we considered that the neurotoxic phenotype observed in scrapie-exposed ovine PrP *Drosophila* was due to prion replication in the fly. To verify if this was the case, we examined scrapie-exposed *Drosophila* for the presence of hallmark features of mammalian prion disease, namely, accumulation of prion-seeding activity, PK-resistant PrP^**Sc**^ and transmissible prions (Oesch et al. 1985; Prusiner 2004).

To assess accumulation of prion-seeding activity, *Drosophila* were exposed at the larval stage to either scrapie-infected or control prion-free sheep brain material. At various time points (≤ 40 days) during their adult lifespan, head homogenate was prepared from groups of flies for use as substrate in PMCA reactions. Prion-seeding activity was only observed in head homogenate prepared from scrapie-infected VRQ PrP *Drosophila* aged ≥ 20 days of age and showed a progressive increase in titre from 20 to 40 days of age (Thackray et al. 2018). In contrast, no prion-seeding activity was detected in PMCA reactions with seed prepared from VRQ PrP *Drosophila* mock infected with control prion-free sheep brain material, or from scrapie-exposed or mock-infected 51D control flies.

The prion-seeding activity observed in scrapie-inoculated VRQ PrP *Drosophila* was associated with the presence of PK-resistant PrP, a pathognomonic marker of prion disease in mammalian hosts (Prusiner 2004). SDS PAGE/western blot detection of PK-resistant PrP in VRQ PrP *Drosophila* showed that this material had a different molecular mass profile to that present in the original PG127 inoculum, which was expected considering the differences in *N*-linked glycosylation that exists between *Drosophila* and mammalian hosts (Marchal et al. 2001; März et al. 1995; Repnikova et al. 2010).

**Fig. 4:**
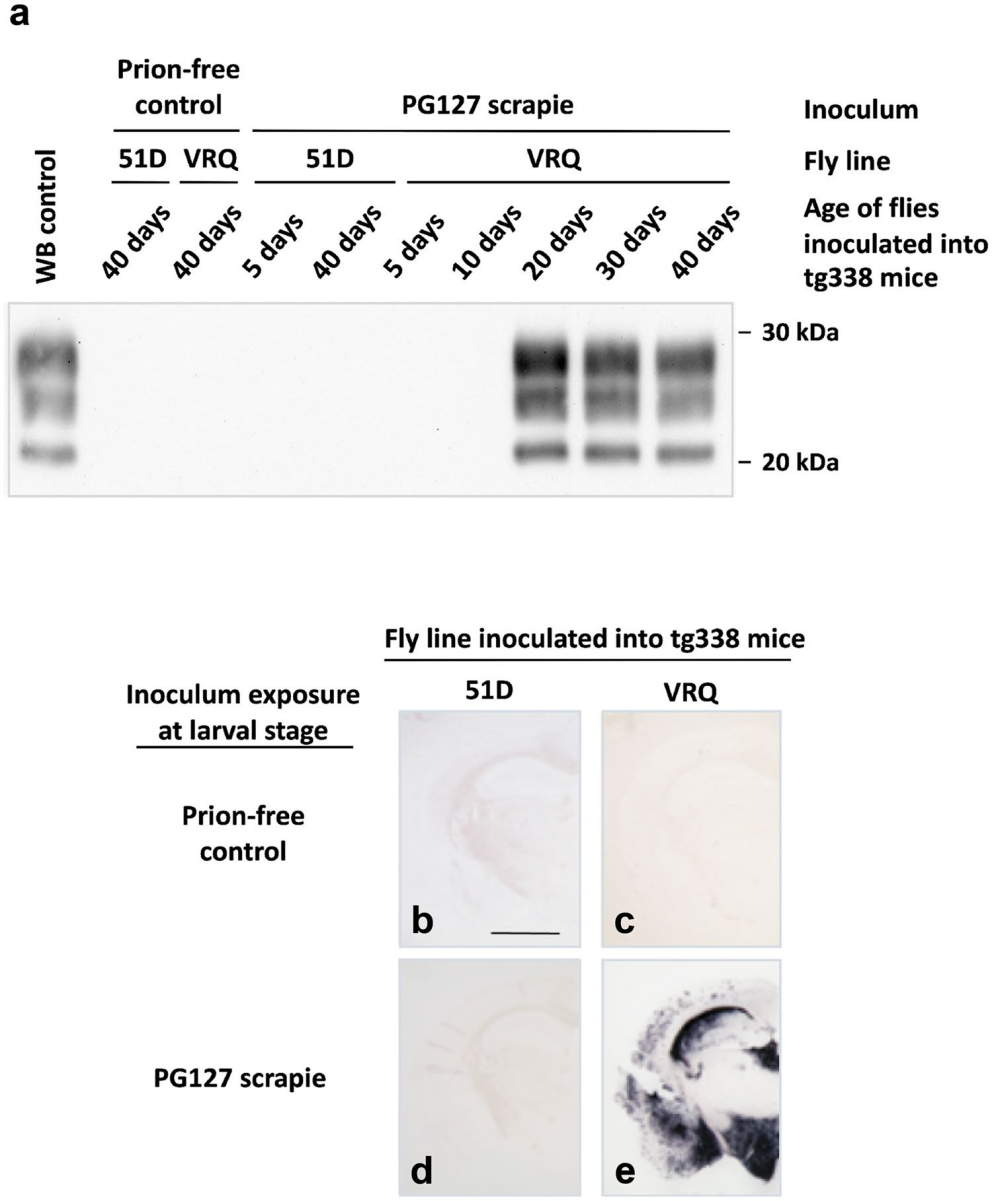
Detection of prion infectivity in scrapie-exposed PrP transgenic *Drosophila. Elav* × VRQ PrP transgenic (VRQ) and *Elav* × 51D (51D) *Drosophila* were exposed at the larval stage to PG127 scrapie-infected or prion-free control sheep brain material. At various times after hatching, head homogenate was prepared from harvested flies and inoculated into tg338 mice. Inoculated mice were euthanised when they showed clinical signs of prion infection or after 250 days for those that did not develop clinical disease. Mice were considered positive for prion disease when PK-resistant PrP27–30 was detected in brain tissue by western blot. (**a**) Western blot detection of PK-resistant PrP27–30 in the brains of tg338 mice with clinical prion disease. Western blot (WB) control is PG127 sheep scrapie material. Molecular mass markers in kDa shown on the right; (**b**–**e**) PET blot analysis of tg338 mouse brains from animals inoculated with 40-day-old *Elav* × 51D (51D) (**b**, **d**) or *Elav* × VRQ PrP transgenic (VRQ) (**c**, **e**) *Drosophila* exposed to prion-free control (**b**, **c**) or PG127 scrapie-infected (**d**, **e**) sheep brain material. Scale bar represents 150 µm. (Modified from Thackray et al., *Brain*, 2018, 141: pp2700-2710)

We performed fly-to-mouse transmission experiments in order to detect bona fide prion infectivity accumulation in scrapie-exposed ovine PrP *Drosophila*. In these experiments, aliquots of the same head homogenate prepared from flies used as seed in the PMCA experiment described above was inoculated into the tg338 mouse line, which is transgenic for VRQ ovine PrP. No prion infectivity was detected in head homogenates prepared from 5 or 10-day-old PG127-exposed VRQ PrP *Drosophila*, while that from 20-day-old flies gave an incomplete attack rate in tg338 mice. In contrast, tg338 mice inoculated with head homogenates prepared from 30 and 40-day-old scrapie-exposed VRQ PrP *Drosophila* developed a 100% attack rate for clinical prion disease transmission, with incubation times of 89 ± 3 and 89 ± 2 days, respectively. Prion disease in mice that showed clinical signs of the condition was confirmed by western blot detection of PK-resistant PrP27-30 as shown by the data in Fig. 4. Furthermore, PET blot analysis of the brains of clinically affected mice showed the typical distribution of PrP^**Sc**^ in PG127 scrapie-inoculated tg338 mice. No evidence of clinical prion disease or abnormal PrP accumulation was observed in mice inoculated with head homogenates prepared from VRQ PrP *Drosophila* exposed to control sheep brain homogenate or from 51D flies inoculated with scrapie-infected or control sheep brain material. This result excludes the suggestion that carry-over of the original sheep inoculum was an explanation for prion infectivity detection in scrapie-exposed VRQ ovine PrP *Drosophila*. The fly-to-mouse transmission experiment was repeated twice using fly head homogenates obtained in two independent experiments (using the same fly lines and the same sheep scrapie prion strain). The incubation times and attack rates we observed were consistent across the three independent experiments (Thackray et al. 2018).

We also investigated whether the prion infectivity that accumulates in VRQ ovine PrP *Drosophila* could be serially propagated in the same fly line (Thackray et al. 2018). To do so, head homogenate from scrapie-infected 30-day-old adult VRQ ovine PrP or 51D *Drosophila* (first passage flies) was inoculated into fresh VRQ ovine PrP *Drosophila* (second passage flies) at the larval stage. Second passage VRQ ovine PrP *Drosophila* were allowed to hatch, and head homogenate prepared from groups of flies euthanised at 5 and 30 days of age was inoculated into tg338 mice in order to assess prion infectivity in these samples. Mice that received head homogenate from 30-day-old second passage VRQ ovine PrP *Drosophila* showed 100% attack rate for clinical signs of mouse prion disease and an incubation period of 89 ± 4 days. The brains of clinically affected mice were characterised by PK-resistant PrP27-30 evidenced by SDS PAGE/western blot and the typical distribution of PrP^**Sc**^ in PG127 scrapie-inoculated tg338 mice shown by PET blot analysis. No clinical signs were observed, and no abnormal PrP accumulated in tg338 mice inoculated with 5-day-old second passage VRQ PrP *Drosophila* head homogenate or from second passage VRQ PrP *Drosophila* exposed to first passage 51D control flies. These results demonstrate that mammalian prions can be serially propagated in VRQ PrP *Drosophila* following experimental inoculation of fly head homogenate. Collectively, these data unequivocally demonstrated that infectious ovine prions replicate and progressively accumulate in scrapie-exposed VRQ ovine PrP transgenic *Drosophila*.

### Preservation of prion strain phenotypes in *Drosophila*

The molecular components and cellular machinery necessary to generate infectious neurotoxic prions remain undefined (Supattapone 2014)**.** PrP^**C**^ is ubiquitously expressed and well conserved in mammalian hosts, and it is possible that this group of species is permissive for prion propagation because they have co-evolved specific co-factors necessary for prion protein misfolding and replication. We tested this hypothesis by determining whether authentic prion propagation occurred in *Drosophila melanogaster*, an invertebrate phylogenetically separated from mammals by millions of years of divergent evolution and that lacks the presence of an orthologous PrP gene in its genome (Rivera-Milla et al. 2006).

**Fig. 5:**
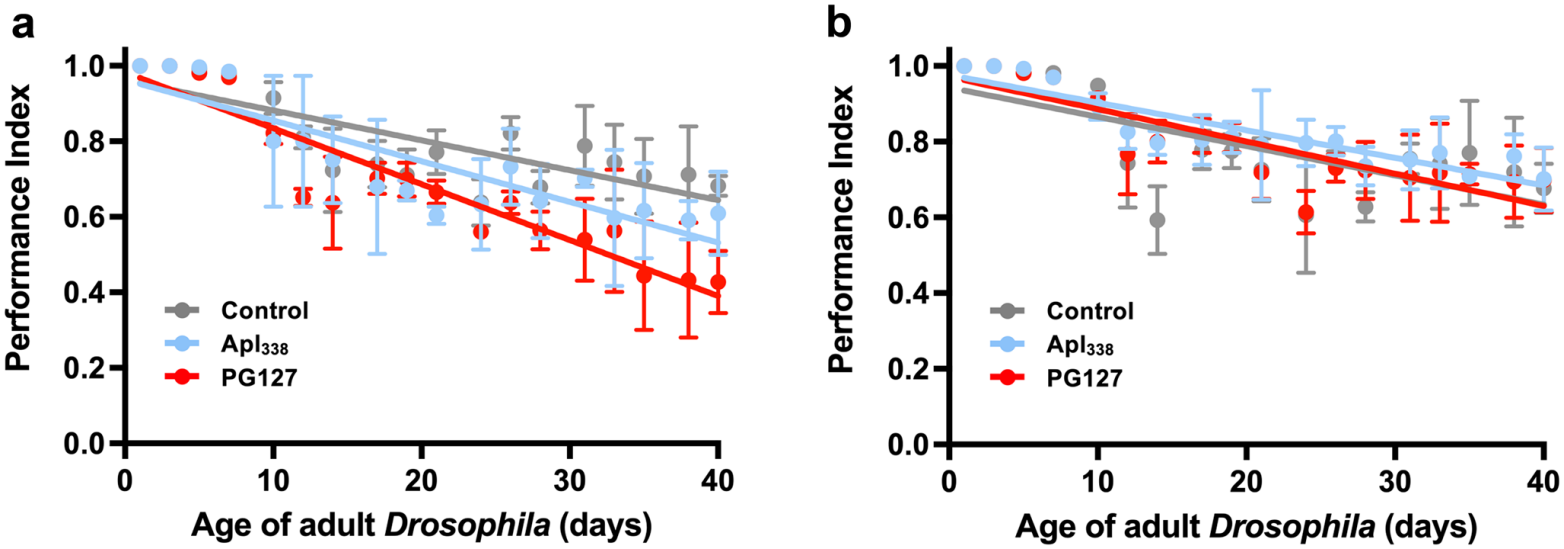
Prion-induced toxicity in scrapie-exposed PrP transgenic *Drosophila.* Adult *Actin* × VRQ ovine PrP (**a**) or *Actin* × control 51D (**b**). *Drosophila* were assessed for their locomotor ability by a negative geotaxis climbing assay following exposure at the larval stage to PG127 (red line), Apl_338_ (light blue line) ovine scrapie strains derived in tg338 mice (VRQ ovine PrP transgenic) or prion-free (grey line) tg338 mouse brain homogenate. The data shown are linear regression plots of the mean performance index ± SD for three groups of flies per time point calculated. (Modified from Thackray et al., *Brain*, 2018, 141: pp2700-2710, supplementary data)

We performed fly-to-mouse transmission experiments in tg338 mice (Thackray et al. 2018) using a panel of ovine prion strains characterised by unique incubation times and PK-resistant PrP27-30 molecular profile following selection in ovine PrP transgenic mice (Thackray et al. 2011, 2012a). Accordingly, we exposed VRQ or ARQ ovine PrP *Drosophila* at the larval stage to ovine prions isolated in VRQ or ARQ ovine PrP mice, respectively. Collectively, these flies were susceptible to all of the ovine prion strains as shown by development of an accelerated decline in locomotor ability of adult flies (Thackray et al. 2018). These data implied that prion strain phenotype was conserved during propagation in PrP *Drosophila*. The severity of the neurotoxic phenotype in VRQ ovine PrP *Drosophila* induced by a ‘short’ incubation time ovine prion strain (tg338-passaged PG127, also known as G_**338**_) was greater than that induced by a ‘long’ incubation time strain (Apl_**338**_) as seen by data in Fig. 5. This implies that the pathogenic determinants of different prion strains manifest themselves in mice and *Drosophila*.

We next used serial passage in tg338 mice to perform comparative prion strain typing studies between each original prion strain isolated in PrP transgenic mice and these prion strains propagated in ovine PrP *Drosophila*. The experiments showed that the disease phenotypes observed in tg338 mice inoculated with the original prion strains were similar to those obtained following inoculation with the equivalent prion strain propagated in ovine PrP *Drosophila* as shown by representative data in Table 1 (Thackray et al. 2018). In addition, the molecular profile of PK-resistant PrP^**Sc**^ and the vacuolar lesion profile in the brains of tg338 mice inoculated with the original ovine prions and those propagated in PrP *Drosophila* were indistinguishable.

**Table 1:** Scrapie strains retain transmission properties after passage in PrP transgenic *Drosophila*

	Original strain isolated in tg338 mice		*Drosophila*-passaged strain isolated in tg338 mice			
			First passage		Second passage	
Scrapie strain	Attack rate	IP	Attack rate	IP	Attack rate	IP
PG127	6/6	61 ± 2	6/6	87 ± 4	6/6	59 ± 1
Apl_338_	6/6	631 ± 83	3/5	642 ± 57	5/5	766 ± 125

The ovine classical scrapie prion strains PG127 and Apl_338_ (isolated in ovine VRQ tg338 mice) were transmitted to tg338 ovine PrP transgenic mice. In parallel, *Actin* × VRQ PrP *Drosophila* were exposed to the PG127 and Apl_338,_ prion strains at the larval stage. Head homogenates were prepared from adult flies aged 30 days and serially transmitted in tg338 mice (two iterative passages) by intracerebral inoculation. Mice were euthanised when they showed clinical signs of prion infection and after 250 days for those that did not develop clinical disease. Mice were considered positive for prion disease when PK-resistant PrP27–30 was detected in brain tissue by western blot. The attack rate (number of prion positive mice/total number of mice inoculated) is reported for each treatment group. The incubation period (IP) for inoculated mice, which represents the mean time from inoculation to euthanasia for each inoculated group of animals, is reported in days ± SD. (Updated from Thackray et al., *Brain*, (2018), 141: pp. 2700–2710)

Collectively, these data demonstrated that the biological properties of distinct ovine prion strains were maintained after their propagation in ovine PrP *Drosophila*. The observations imply that molecular and cellular co-factors necessary for strain-specific prion propagation are conserved between phylogenetically diverse species and are not unique to mammalian hosts.

### Sensitive detection of mammalian prions by *Drosophila*

We compared the sensitivity of PrP *Drosophila* to detect mammalian prions to that achieved by mice, the most commonly used mammalian species for this purpose, and to that obtained with in vitro PMCA.

We first compared the sensitivity of VRQ ovine PrP *Drosophila* to detect sheep scrapie prion infectivity and the tg338 mouse line, which is also transgenic for VRQ ovine PrP. To do so, the locomotor ability of adult VRQ ovine PrP *Drosophil*a was assessed by a negative geotaxis climbing assay after exposure at the larval stage to a dilution series of classical scrapie-infected sheep brain homogenate (Thackray et al. 2016). A significant decline in adult VRQ ovine PrP *Drosophila* locomotor ability was induced by dilutions of prion inoculum in the range 10^−2^–10^−10^, which diminished upon exposure to increasing dilution of prion inoculum. In contrast, the tg338 mouse line only detected dilutions of the same prion inoculum in the range from neat to 10^−**5**^ (Andreoletti et al. 2011). These observations showed that VRQ ovine PrP *Drosophila* has a greater sensitivity for ovine prions compared to tg338 mice.

We next determined the sensitivity of bovine PrP *Drosophila* to detect classical BSE prion infectivity. We first used PMCA to detect prion-seeding activity in adult *Drosophila* exposed to dilutions of classical BSE inoculum at the larval stage (Thackray et al. 2021). Prion-seeding activity was detected in head homogenate prepared from classical BSE-exposed bovine PrP *Drosophila* aged ≥ 20 days, and the sensitivity of detection progressively increased as the flies aged. For example, prion-seeding activity was detected in head homogenate of 20-day-old adult *Drosophila* after larval exposure to ≤ 10^−8^ dilution of classical BSE-infected bovine brain material, while 40-day-old flies were PMCA-positive after exposure to ≤ 10^−14^ dilution of the same inoculum. We also assessed the sensitivity of bovine PrP *Drosophila* for classical BSE prion detection by way of negative geotaxis and survival assays. After hatching, adult bovine PrP *Drosophila* showed accelerated decline in climbing ability compared to control treated flies, which became progressively more severe with age but decreased with increasing dilution of classical BSE inoculum. A significant response was seen following inoculation with ≤ 10^−12^ dilution of classical BSE-infected bovine brain material.

**Fig. 6:**
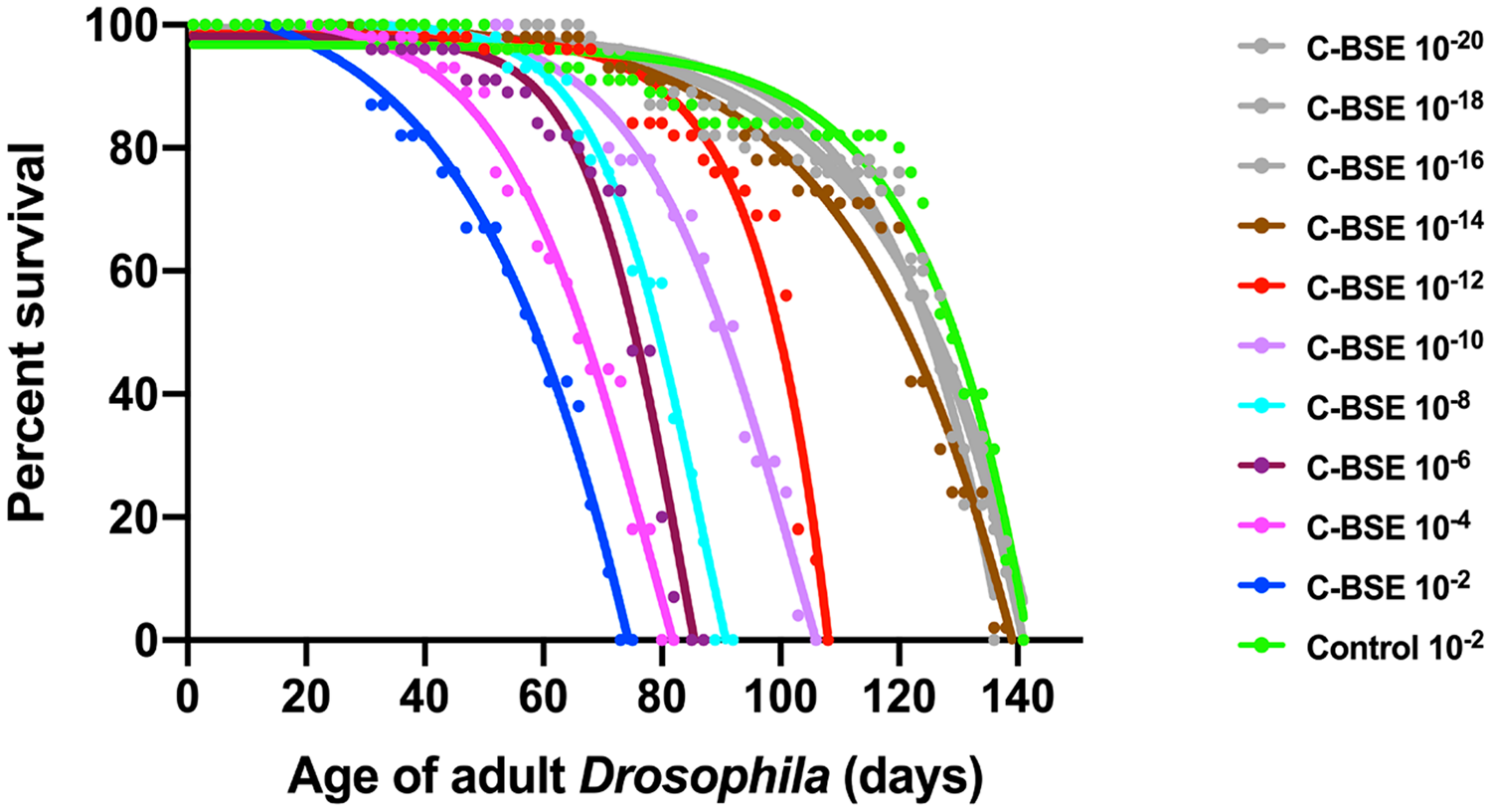
Accelerated loss of survival of classical BSE-exposed bovine PrP *Drosophila.* Adult *Elav* × bovine PrP *Drosophila* were assessed for survival following exposure at the larval stage to 10^−2^–10^−20^ dilutions of classical BSE-infected bovine brain material. Control inoculum was a 10^−2^ dilution of prion-free bovine brain material (control 10^−2^). The number of surviving flies was recorded three times a week and the data shown as Kaplan–Meier plots. C-BSE = classical BSE. (Thackray et al., *J. Biol. Chem.*, 2021, 297, 100,878)

The median survival time of bovine PrP *Drosophila* exposed to classical BSE-infected bovine brain homogenate was reduced compared to the response seen after exposure to control prion-free inoculum, which progressively lengthened upon exposure to increasing dilution of inoculum as shown by the data in Fig. 6. Significant accelerated loss of survival by adult bovine PrP *Drosophila* was observed after exposure of larvae to ≤ 10^−12^ dilution of classical BSE-infected bovine brain material. Collectively, these different assays show that the limit of sensitivity of bovine PrP *Drosophila* for detection of classical BSE was in the range of 10^−12^–10^−14^ dilution of bovine prion-infected brain material (Thackray et al. 2021).

We subsequently established the level of sensitivity of bovine PrP transgenic mice and in vitro PMCA for the same classical BSE inoculum used to infect bovine PrP *Drosophila* (Thackray et al. 2021). When a dilution series of the classical BSE inoculum was inoculated intracerebrally into tg110 bovine PrP transgenic mice (Castilla et al. 2003), positive transmission was observed in animals exposed to ≤ 10^−4^ dilution of bovine prion-infected brain material. A second dilution series of the classical BSE isolate was used as seed in PMCA reactions that were subsequently tested for the presence of PK-resistant PrP^**Sc**^. Positive PMCA activity was observed in reaction tubes seeded with dilutions ≤ 10^−7^ of the classical BSE isolate. Collectively, these data reveal that bovine PrP *Drosophila* are 10^6^-fold more sensitive in identifying classical BSE compared to the bovine PrP mouse bioassay and 10^5^-fold more sensitive than PMCA.

For appropriate biosecurity, studies with BSE, chronic wasting disease (CWD) or CJD-inoculated *Drosophila* require the flies to be maintained at containment level 3 for prion research, an enhanced level of safety above the containment level 2 used for the sheep scrapie studies. In this manner, PrP transgenic *Drosophila* can be used to begin to address important questions on the pathogenic potential of known and possible zoonotic prions such as CWD in cervids.

### Bioassay of prion-infected blood plasma in *Drosophila*

Our finding that PrP *Drosophila* showed a high sensitivity for detection of mammalian prions led us to speculate that this invertebrate host could detect the apparent low titre of prion infectivity in the blood of prion-diseased animals. In order to test this hypothesis, we used VRQ ovine PrP *Drosophila* to bioassay known prion-positive blood samples from VRQ PrP homozygous sheep experimentally infected with classical scrapie (Thackray et al. 2016). We chose to bioassay sheep plasma as this blood fraction is reported to contain a low level of prion infectivity and is notoriously difficult to assess by conventional prion bioassay (Lacroux et al. 2012).

Ovine PrP *Drosophila* were exposed at the larval stage to a dilution series of plasma isolated from a sheep with experimental clinical scrapie disease, and the locomotor ability of adult *Drosophila* was assessed by a negative geotaxis climbing assay (Thackray et al. 2016). Ovine PrP *Drosophila* demonstrated an accelerated decline in locomotor ability soon after hatching following larval exposure to scrapie-infected sheep plasma compared to the response seen after exposure to scrapie-free sheep plasma. The rate of decline in locomotor ability diminished with exposure to increasing dilution of scrapie-infected plasma, indicative of titration of a particulate transmissible moiety, a characteristic feature of the infectious scrapie agent (Stamp 1962). Strikingly, the limit of detection in ovine PrP *Drosophila* was a dilution of ≤ 10^−6^ of plasma from sheep with experimental-induced clinical scrapie.

We next investigated whether PrP transgenic *Drosophila* could detect plasma from sheep with natural scrapie. The donor animals were VRQ PrP homozygous sheep born and maintained in a flock endemic for natural classical scrapie (Dexter et al. 2009). Blood samples were collected from sheep shortly after birth and then at regular intervals until the animals showed clinical signs of terminal scrapie disease when they were euthanised. All of the euthanised blood donor sheep were shown to be positive for scrapie disease by routine testing for disease-associated PrP, and the mean survival time of the animals was 695 ± 25 days. We assessed the locomotor ability of adult *Drosophil*a, transgenic for a cytoplasmic form of VRQ ovine PrP [VRQ(cyt)] by a negative geotaxis climbing assay after larval exposure to plasma from sheep with natural scrapie (Thackray et al. 2016).

Adult VRQ(cyt) PrP transgenic *Drosophila* showed no accelerated decline in locomotor activity after exposure at the larval stage to plasma prepared from natural scrapie-affected sheep aged 3 months compared to that seen following exposure to control plasma. However, a significant response was seen with plasma from donor animals aged ≥ 6 months that was most pronounced in terminal stage disease samples. These observations demonstrated the ability of PrP transgenic *Drosophila* to detect prion-infected plasma samples from asymptomatic scrapie-infected sheep since clinical signs of the disease were not evident until these animals were ≥ 20 months of age (Thackray et al. 2016).

### Transcriptional signature of prion-induced neurotoxicity in *Drosophila*

An important goal in prion biology is to establish the mechanism of prion-induced neurotoxicity as this will underpin therapeutic and control strategies for human and animal prion diseases. Despite significant advances in this area (Hwang et al. 2009; Moreno et al. 2012; Scheckel et al. 2020; Sorce et al. 2020; Vincenti et al. 2015), understanding this process remains to be fully defined. We reasoned that *Drosophila*, a genetically well-defined animal model, provided a novel system to probe the identity of cellular and molecular pathways associated with prion-induced neurotoxicity.

Accordingly, we performed RNA-Seq-based transcriptome analysis on 5 and 40-day-old adult fly head homogenate prepared from VRQ ovine PrP *Drosophila* and the control 51D fly line after larval exposure to either scrapie-infected or control prion-free sheep brain homogenate (Thackray et al. 2020). Following prion exposure at the larval stage, 5-day-old adult VRQ ovine PrP *Drosophila* lack a significant locomotor defect, while 40-day-old adult flies showed an accelerated decline in locomotor activity (Thackray et al. 2018), and therefore represent pre-clinical and clinical time points, respectively.

Collectively, a total of 9672 *Drosophila* genes were detected in prion-infected and control fly samples harvested at 5 days and 40 days post hatching (Thackray et al. 2020). This represented expression of > 61.7% of the *Drosophila melanogaster* genome during the course of the experiment. Temporal and quantitative differentially expressed gene expression changes were seen in prion-exposed PrP *Drosophila* that were distinct from those in similarly treated 51D flies. This implied that the gene changes seen in scrapie exposed ovine PrP *Drosophila* were prion-induced and not simply an effect of these flies ageing. We performed pathway enrichment analysis using combined lists of differentially expressed genes in prion-exposed PrP *Drosophila* on both days 5 and 40 in order to obtain unbiased predictions of cellular and biochemical events perturbed as a consequence of prion exposure (Thackray et al. 2020). We subsequently inspected the overrepresented pathways for genes either up- or down-regulated at different time points in order to predict when particular cellular functions were enhanced or suppressed following prion infection in the fly.

Up-regulation of genes involved with cell cycle activity and DNA damage response (DDR) was identified in 5-day-old prion-exposed adult PrP *Drosophila* after larval exposure to scrapie material. At this time point, top-ranked up-regulated functions were cell cycle activity and DNA damage regulation, together with functions that control cell cycle progression, namely GADD45 and ataxia telangiectasia mutated (ATM) signalling (Liebermann and Hoffman 2008). Usually, post mitotic neurons do not participate in cell cycle activity as this is considered to be detrimental to these cells (Frank and Tsai 2009). In this context, post mitotic neurons may have the potential to revert to a de-differentiated state, which might be linked to activation of apoptotic pathways and concomitant neurodegeneration (Arendt 2001; Arendt et al. 2000; Herrup and Yang 2007).

Down-regulation of eIF2 signalling gene expression was identified in 40-day-old ovine PrP *Drosophila*, previously exposed to scrapie material at the larval stage. Prion-exposed PrP *Drosophila* at this time point showed a dramatic down-regulation of multiple genes of the eIF2 signalling pathway, including those that encoded 40S and 60S ribosomal proteins or genes that encoded components of various translational initiation cofactors, including eIF1, eIF2, eIF3 and eIF4. Since eIF2 signalling is a major regulator of initiation of mRNA translation (Kapur et al. 2017), down-regulation of this pathway was indicative of suppression of protein synthesis in prion-exposed *Drosophila*. Neurons are highly dependent upon sustained and efficient mRNA translation in order to undergo two important processes, namely neurotransmitter release and synaptic plasticity.

The integrated stress response (Pakos-Zebrucka et al. 2016) or the unfolded protein response (Ron 2002) is an important regulator of eIF2 signalling. This stress response inhibits general protein synthesis through eIF2α phosphorylation, and subsequent sequestration of the eIF2α guanine nucleotide exchange factor eIF2B. The fly gene *Gcn2*, which encodes a kinase that phosphorylates eIF2α, was up-regulated in prion-exposed PrP *Drosophila*, which suggests activation of the integrated stress response in these flies. This observation correlates with studies in cells (Roffe et al. 2010) and mice (Moreno et al. 2012) that show that prion disease progression triggers activation of the PERK/eIF2α branch of the unfolded protein response with its subsequent block on protein translation.

A second major signalling pathway that displayed differential gene expression in prion-exposed PrP *Drosophila* was the mTOR signalling pathway (Thackray et al. 2020). mTOR mediates an important role in the regulation of autophagy, a process that serves to prevent the accumulation of aberrantly toxic protein aggregates or organelles (Abdelaziz et al. 2019). We found that 40-day-old prion-exposed PrP *Drosophila* were characterised by marked changes in gene expression for some genes of the mTOR pathway. In addition to down-regulated eIF and ribosomal subunit genes, several genes were up-regulated including *Dsor1* (Tsuda et al. 1993); *Lk6*, a serine/threonine-protein kinase that interacts with mitogen-activated protein kinase 1 MAPk1/ERK2 (Cargnello and Roux 2011) and Pkc98E, a regulatory protein kinase activated by diacylglycerol or Ca^**2+**^ (Lipp and Reither 2011).

Mitochondrial dysfunction was identified by Ingenuity Pathway Analysis as the top toxicity pathway in prion-exposed *Drosophila*. It was found that prion-exposed 40-day-old adult VRQ ovine PrP *Drosophila*, exposed to scrapie at the larval stage, were characterised by down-regulation of genes encoding principal components of the electron transport chain including NADH dehydrogenase, ATP synthase and various anti-oxidants including superoxidase dismutase-2 and glutaredoxin 1. Neurons are vulnerable to mitochondrial dysfunction because of the need to satisfy the large energy demands of synaptic development, transmission and plasticity. Mitochondrial dysfunction is a feature of hamster (Choi et al. 1998) and mouse (Keller et al. 2019; Siskova et al. 2010) models of prion disease. In addition, down-regulation of mRNA and protein levels of mitochondrial proteins have been reported in post-mortem tissues of Creutzfeldt-Jakob disease (CJD) patients (Ansoleaga et al. 2016).

**Fig. 7:**
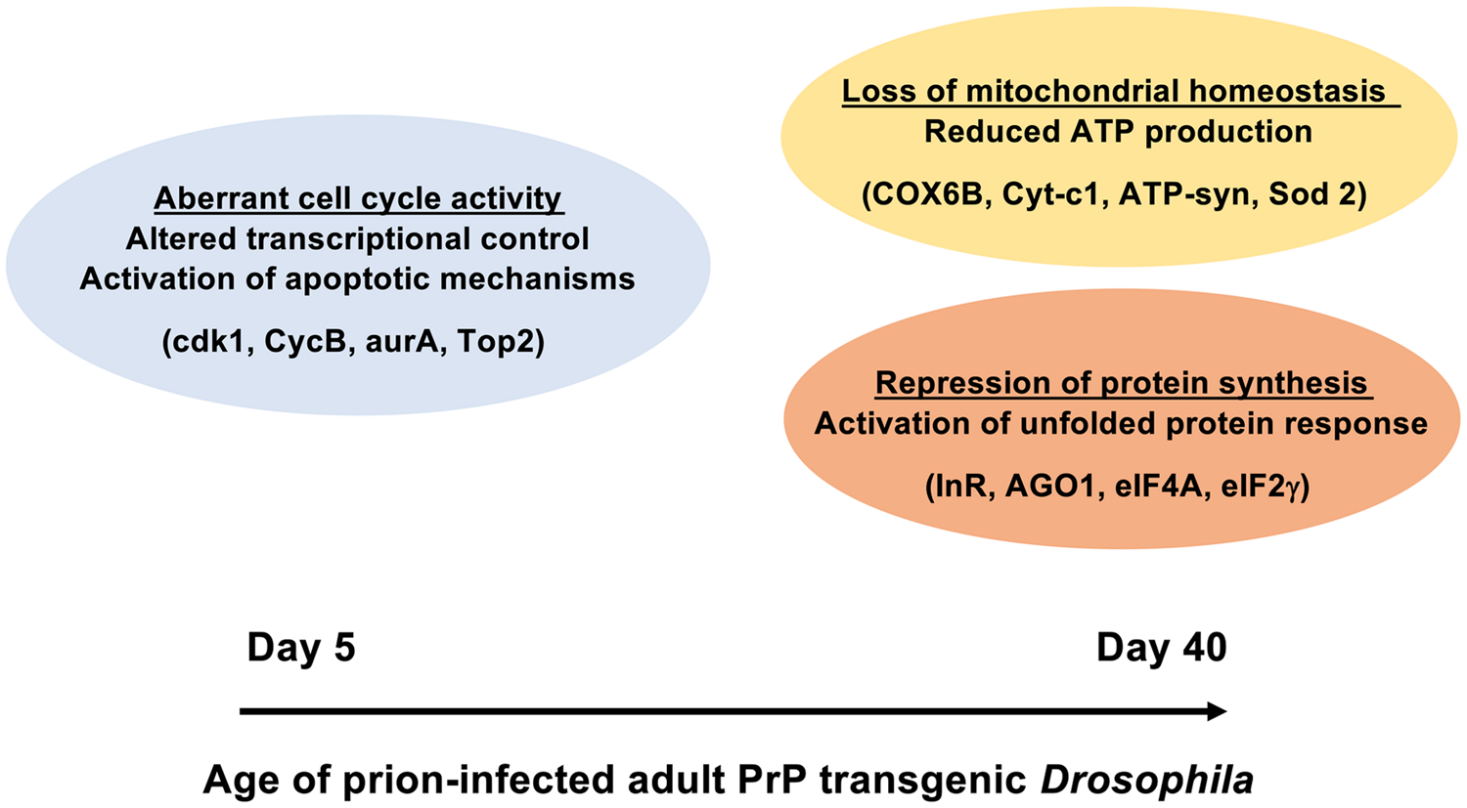
Model for prion-induced neurotoxicity in PrP transgenic *Drosophila.* Proposed major cellular events, together with representative participating genes (shown in brackets), associated with prion-induced neurotoxicity in PrP transgenic *Drosophila*. (Modified from Thackray et al., *Biochem. J.*, 2020, 477: pp833–852)

Based on these observations, we speculate that following initial prion infection at the larval stage, subsequent PrP^**Sc**^ accumulation within *Drosophila* neurons has an adverse effect upon critical cellular processes, that in turn leads to a genotoxic effect with resultant dysregulated gene expression and aberrant cell cycle activity coupled with the activation of apoptotic mechanisms (Thackray et al. 2020). We further speculate that these early prion-induced events drive a loss of neuronal mitochondrial homeostasis and a repression of protein synthesis as neurons attempt to accommodate the cellular stress associated with PrP misfolding, which is summarised in Fig. 7. We are unable to differentiate whether the proposed loss of mitochondrial homeostasis, which will invariably be accompanied by a reduction in ATP production, is the stimulus for repression of protein synthesis or whether loss of protein synthesis drives loss of normal mitochondrial status. However, our use of *Drosophila*, a genetically well-defined tractable experimental host amenable to silencing and overexpression of specific genes, will allow us to begin to attempt to differentiate between these possibilities.

## Concluding comments

We have shown that PrP transgenic *Drosophila* provide paradigm shift in the bioassay of transmissible mammalian prions. This novel invertebrate animal model shows robust and extremely sensitive detection of mammalian prion infectivity in a reasonably high-throughput and rapid, cost-effective manner. The facile nature of transgenesis in *Drosophila* renders this host amenable to the generation of flies transgenic for different species forms of mammalian PrP by tractable and timely means, in order to bioassay prions from a wide variety of prion diseases. This is particularly relevant given the emergence of new prion diseases in animals, such as camel prion disease, together with the new reservoirs of CWD in cervids that pose fresh challenges to human food safety since their zoonotic potential is unknown. Accordingly, more prion bioassays will be required to fully determine the relative risk these new prion disease occurrences present to the human food chain and PrP transgenic *Drosophila* provide the ideal host for this role.

Given the infectious nature of the transmissible prion agent, sensitive detection of this pathogen is required to characterise the spread of these diseases within affected populations. The detection of mammalian prions by PrP transgenic *Drosophila* was more sensitive than that achieved by in vivo bioassay in mice and in vitro PMCA. PrP transgenic *Drosophila* were sufficiently sensitive to be able to detect prion-infected blood from sheep with pre-clinical scrapie disease and in a timeframe significantly shorter than that achievable by bioassay in mammalian species, including transfusion in the natural host. This provides the basis for development of a much needed versatile and efficient blood test for mammalian prion disease based on the use of PrP transgenic *Drosophila*.

Significantly, we have shown that mammalian prions propagate faithfully in PrP transgenic *Drosophila* with preservation of prion strain phenotype. This highlights that the cellular and molecular components required for prion protein misfolding and prion propagation are maintained between *Drosophila* and mammalian species, despite the fact that this invertebrate does not normally express PrP. This in turn has allowed the cellular processes and molecular components associated with prion-induced neurotoxicity to be investigated in the fly through RNASeq analysis. This approach revealed candidate pathways and processes, e.g. eIF2 signalling and mitochondrial dysfunction, that may function both as biomarkers of prion infection, and potential therapeutic targets. The extensive molecular and transgenic repertoire of *Drosophila* will allow candidate genetic modifiers of prion-induced neurotoxicity to be further validated through RNAi-mediated gene silencing or transgenic overexpression during prion infection in PrP *Drosophila*.

Overall, we have established PrP transgenic *Drosophila* as a new animal system that will make a significant contribution to the understanding of mammalian prion biology.
